# Using threshold analysis to assess the robustness of public health intervention recommendations from network meta-analyses: application to accident prevention in households with children under five

**DOI:** 10.1186/s12889-022-13377-5

**Published:** 2022-05-13

**Authors:** Molly Wells, Sylwia Bujkiewicz, Stephanie J. Hubbard

**Affiliations:** Biostatistics Research Group, University of Leicester, Leicester, UK

**Keywords:** Meta-analysis, Network meta-analysis, Threshold analysis, Risk of bias, Bias adjustment, Evidence synthesis, Public health

## Abstract

**Background:**

In the appraisal of clinical interventions, complex evidence synthesis methods, such as network meta-analysis (NMA), are commonly used to investigate the effectiveness of multiple interventions in a single analysis. The results from a NMA can inform clinical guidelines directly or be used as inputs into a decision-analytic model assessing the cost-effectiveness of the interventions. However, there is hesitancy in using complex evidence synthesis methods when evaluating public health interventions. This is due to significant heterogeneity across studies investigating such interventions and concerns about their quality.

Threshold analysis has been developed to help assess and quantify the robustness of recommendations made based on results obtained from NMAs to potential limitations of the data. Developed in the context of clinical guidelines, the method may prove useful also in the context of public health interventions. In this paper, we illustrate the use of the method in public health, investigating the effectiveness of interventions aiming to increase the uptake of accident prevention behaviours in homes with children aged 0–5.

**Methods:**

Two published random effects NMAs were replicated and carried out to assess the effectiveness of several interventions for increasing the uptake of accident prevention behaviours, focusing on the safe storage of other household products and stair gates outcomes. Threshold analysis was then applied to the NMAs to assess the robustness of the intervention recommendations made based on the results from the NMAs.

**Results:**

The results of the NMAs indicated that complex intervention, including *Education, Free/low-cost equipment, Fitting equipment and Home safety inspection,* was the most effective intervention at promoting accident prevention behaviours for both outcomes. However, the threshold analyses highlighted that the intervention recommendation was robust for the stair gate outcome, but not robust for the safe storage of other household items outcome.

**Conclusions:**

In our case study, threshold analysis allowed us to demonstrate that there was some discrepancy in the intervention recommendation for promoting accident prevention behaviours as the recommendation was robust for one outcome but not the other. Therefore, caution should be taken when considering such interventions in practice for the prevention of poisonings in homes with children aged 0–5. However, there can be some confidence in the use of this intervention in practice to promote the possession of stair gates to prevent falls in homes with children under 5. We have illustrated the potential benefit of threshold analysis in the context of public health and, therefore, encourage the use of the method in practice as a sensitivity analysis for NMA of public health interventions.

## Background

Evidence synthesis methods, including systematic reviews and meta-analysis, are used in evidence-based decision-making, for example, carried out as part of the technology appraisals of new health interventions. A range of meta-analytic methods are available for different data scenarios. Pairwise meta-analysis pools evidence from multiple studies that compare head-to-head two interventions, that are the same or similar across studies, to gain a pooled overall estimate of the relative treatment effect. However, issues with pairwise meta-analysis arise when more than two interventions need to be compared. Network meta-analysis (NMA) expands on the pairwise meta-analysis framework by allowing for the comparison of multiple interventions in a single analysis. The results from a NMA are often used to inform a decision-analytic model assessing the cost-effectiveness of the interventions [1]. The effectiveness and cost-effectiveness of interventions are vital components in policy decision-making and the development of guidelines, for example, by the National Institute for Health and Care Excellence (NICE).

Despite the known benefits of NMA, there is some hesitancy in using NMA methods in public health intervention appraisals. Public health interventions can be highly complex as they can consist of multiple and often overlapping components. It is common to see substantial between-studies heterogeneity due to, for example, different study designs, which is often listed as the reason for not using meta-analysis methods [2].

As well as substantial between-studies heterogeneity, there is often concern regarding the quality of the studies evaluating public health interventions. Due to the nature of public health outcomes and corresponding interventions, there tends to be a broader range of study types in contrast to individual-focused randomised controlled trials (RCTs) typically seen in clinical settings. Due to the nature of RCTs, particularly the randomisation and blinding, they are considered to be the least biased source of evidence compared to other study designs such as non-randomised controlled trials (NRCTs) and observational studies. The broad range of study designs in public health introduces issues with the validity of the results from these studies and increases the potential risk of bias. This is one of the reasons behind the hesitancy for using NMA methods in the public health setting.

A systematic review by Achana et al. (2014) [3], concluded that complex evidence synthesis methods should be considered and used more in the appraisal of public health interventions to aid decision-makers and to make the evaluations more useful. This review highlighted that, of the 39 NICE public health appraisals published between 2006 and 2012, only 9 (23%) used pairwise meta-analyses for the evaluation of the interventions, and only one appraisal conducted a network meta-analysis. The main reasons for not using more complex evidence synthesis methods were stated to be due heterogeneity in outcomes, methods and interventions [3]. An update of the review of methods used in NICE public health appraisals by Smith et al. (2021) [2], highlighted that there is increasing use of evidence synthesis methods in the appraisals of public health interventions by NICE. Thirty-one percent (14/45) of NICE public health intervention appraisals used a meta-analysis as part of the statistical analysis assessing the effectiveness of such interventions, which is an increase of 8% since 2012. However, only one of these appraisals conducted a NMA, this highlights the limited use of such methods in public health intervention appraisals despite the known benefits [2].

All studies included in a NMA should be assessed in terms of their quality and the potential risk of bias. If the studies included in the NMA have issues with their conduct and design, causing problems with their validity or their relevance, then there will be concerns regarding the reliability and validity of the NMA estimates and rankings. The Cochrane risk of bias tool can be used to assess the quality and potential risk of bias for individual studies [4]. This is typically used for RCTs where the studies are assessed on several aspects whereby possible bias could occur. Each aspect of the trial design that could introduce bias is then assigned a judgment based on how susceptible the study is to bias. These judgements are rated “high”, “low”, or “unclear” [5].

Threshold analysis, a method recently proposed by Phillippo et al. [4], quantifies the sensitivity of effect estimates and decisions resulting from a NMA to any changes in the evidence that could be due to imprecision in the effect estimates or potential bias. In this paper, we aim to illustrate that the application of threshold analysis in the public health setting can allow researchers and policy makers to assess and quantify the credibility of the results from NMAs in the presence of evidence that could be at risk of bias. We illustrate this using two examples of already published NMAs investigating the effectiveness of interventions to increase the uptake of accident behaviours in homes with children under 5.

## Methods

### Network meta-analysis

Network meta-analysis (NMA) allows for the comparison of multiple interventions in a single analysis to obtain the relative effectiveness of all interventions compared to each other. In NMA, the structure of the network is used to gain indirect estimates of effects between interventions that have not been compared directly. For example, by combining trials that have direct evidence comparing interventions B versus A and trials of C versus B, we can estimate the indirect relative effect of interventions C versus A. The use of indirect evidence is suitable provided that we can assume the consistency in the network, indicating that there is little difference between the direct evidence from trials (in this case, trials of C versus A, if they exist in the network) and indirect evidence obtained from the network. By combining the direct and indirect evidence, NMA allows for the estimation of relative intervention effects for all interventions in the network and enables ranking of the interventions according to the probability of an intervention being the best, thus identifying the most effective intervention [6, 7]. The results from the NMA are often incorporated into a decision-analytic model to consider the cost-effectiveness of interventions. We replicated two published NMAs by Achana et al. 2015 [8] and Hubbard et al. 2015 [9] in WinBUGS 1.4.3 using a Bayesian approach which gave effect estimates as odds ratios with 95% credible intervals.

### Threshold analysis

Threshold analysis identifies how sensitive the intervention recommendations based on a NMA are to the smallest changes to the effect estimates that would result in a different optimal intervention being recommended [4, 6]. Rather than adjusting for potential biases, the method derives bias adjustment thresholds to establish the degree to which evidence could change without altering the intervention recommendation. Threshold analysis requires a clear decision rule from which the intervention recommendation is made. The optimal intervention is decided based on which intervention achieves the highest expected intervention effect for the defined outcome (for example, log odds of success) [4, 6]. Positive and negative bias adjustment thresholds form decision invariant bias adjustment intervals. Any changes in the point estimate, due to a bias, that are within the invariant interval will not result in a change of the recommendation. However, if, for example, a confidence or credible interval of an effect estimate in a given study is large, extending beyond the invariant interval, then the intervention recommendation may not be robust due to the imprecision of such estimate. Whereas, if the confidence or credible interval lies within the invariant interval, then this means that the intervention decision for that estimate is robust.

Threshold analysis can be conducted at the study level and the contrast level. Study level threshold analysis considers the impact of any change in the effect estimates from individual studies in the network that could be due to any potential bias, on the results of the NMA, including intervention ranking. Study level threshold analysis helps to assess the robustness of the intervention recommendation based on each study individually. Contrast level threshold analysis examines the robustness of the results from the NMA in the combined evidence for each intervention contrast in the network. That is, assuming that direct evidence for the contrast is present in the network, we assess the impact of any potential bias in the combined evidence for that particular contrast on the results from the NMA. Contrast level analysis is more useful in guideline development as the robustness of the entire body of evidence is considered, rather than just the individual studies [4, 6]. For the full algebraic breakdown of both study and contrast level threshold analyses, refer to Philippo et al. 2018 [4]. The threshold analyses was conducted in RStudio using the package “nmathresh” created by Phillippo et al. 2018 [4].

### Application

We adapted the threshold analysis code to allow for the modelling of a random effects NMA with a binary outcome and applied it to two published NMAs. The NMAs, in the area of accident prevention in homes with children under five, evaluated interventions to increase the uptake of accident prevention behaviours and equipment to prevent poisonings [8] and falls [9].

The data for each NMA were obtained from primary studies identified in separate systematic reviews [10, 11]. We replicated the published NMA using a random effects NMA with a binary outcome, with binomial likelihood, logit link, and vague priors for intervention effects. The outcome of interest for both NMAs was the uptake of accident prevention behaviours and equipment and we were interested in the most effective intervention at increasing the uptake of these behaviours. In this paper, we focus on two outcomes, interventions to promote the safe storage of other household products and possession of a fitted stair gate. Details of the studies included in the networks for each outcome can be seen in Table 1 and Table 2 respectively. For the safe storage of other household products outcomes, there were 15 primary studies assessing the effectiveness of 7 interventions. The studies included 10 RCTs, two NRCTs, two cluster RCTs and one cluster NRCT. Whereas, for the possession of a fitted stair gate outcome, there were 12 studies assessing the effectiveness of 7 interventions. The studies included 10 RCTs and 2 NRCTs. As described in Achana et al. [8] and Hubbard et al. [9], clustering and the use of NRCTs was adjusted for in the NMAs. The quality of the primary studies included in the systematic review were assessed using the Cochrane Collaboration’s risk of bias tool and Newcastle–Ottawa scale for experimental and controlled observational studies, respectively [10, 11].

**Table 1:** Details of studies included in NMA for the safe storage of other household products outcome

Intervention Comparison	Study Number	Study	Study quality and Risk of Bias	Safe storage of other household products/Total number of households
Usual care (1) vs Education (2)	1	Kelly (1987), RCT, USA	A = U,B = Y,F = N	43/54 49/55
	2	Nansel (2002)^a^, RCT, USA	A = Y,B = U,F = Y	65/89 66/85
	3	McDonald (2005), RCT, USA	A = Y,B = U,F = N	3/57 6/61
	4	Gielen (2007), RCT, USA	A = Y,B = N,F = Y	44/62 57/73
	5	Nansel (2008), Non-RCT, USA	A = U,B = N,F = N	59/73 117/144
Usual care (1) vs Education + Free/low cost Equipment (3)	6	Woolf (1992), Cluster-RCT, USA	A = U,B = Y,F = N	60/151 89/150
	7	Clamp (1998), RCT, UK	A = U,B = N,F = Y	49/82 59/83
Usual care (1) vs Education + Equipment + Home Safety inspection (4)	8	Kendrick (1999), Cluster non-RCT, UK	B = N,F = N,C = Y	317/367 322/363
	9	Swart (2008), Non-RCT, South Africa	A = U,B = Y,F = Y	46.86/57.96^b^ 50.87/58.27^b^
	10	Hendrickson (2002), USA, RCT	A = N,B = N,F = Y	14/40 34/38
Usual care (1) vs Education + Equipment (5)	11	Watson (2005), Cluster-RCT, UK	A = Y,B = N,F = Y	327/669 368/693
Education (2) vs Education + Equipment (3)	12	Posner (2004), RCT, USA	A = Y,B = Y,F = N	22/47 34/49
Education (2) vs Education + Equipment (5)	13	Sznajder (2003), RCT, France	A = Y,B = N,F = Y	32/41 40/48
Education + equipment (3) vs Equipment only (7)	14	Dershewitz (1977), RCT, USA,	A = U,B = Y,F = N	1/101^c^ 0/104^c^
Education + Equipment + home Safety inspection (4) vs Education + equipment + home safety inspection + Fitting (6)	15	King (2001), RCT, USA	A = Y,B = Y,F = Y	261/469 273/482

Last column includes the number of households with safe storage out of the total number of households

*Abbreviations*:

1.*A* Adequate allocation concealment, *B* Blinded outcome assessment, *C* The prevalence of confounders does not differ by more than 10% between treatment arms, *CBA* Controlled before-and-after study, *F* At least 80% of participants followed up in each arm, *NMA* Network meta-analysis, *RCT* Randomised clinical trial, *U* Unclear, *Y* Yes, *N* No, *NR* Not reported/not relevant

2.^a^Two intervention arms were combined (tailored advice and tailored advice + care provider feedback)

3.^b^Figures adjusted for the effect of clustering using ICC and method reported in Achana et al. (2015) [8]

4.^c^Continuity correction applied by adding 0.5 and 1 to denominator and numerator to account for the zero events reported (no households that were assessed safely stored other household products)

**Table 2:** Details of studies included in NMA for the possession of fitted stair gates outcome

Intervention Comparison	Study Number	Study	Study quality and Risk of Bias	Number of stair gates/Total number of households
Usual care (1) vs. Education (2)	1	Nansel (2002), RCT	A = U,B = Y,F = N	70/89 76/85
	2	Kendrick (2005), RCT	A = Y,B = U,F = Y	348.44/436.80^a^ 310.93/376.78^a^
	3	Nansel (2008), Non-RCT	A = Y,B = U,F = N	29/38 60/69
Usual care (1) vs. Education + Low/free equipment (3)	4	Clamp (1998), RCT	A = Y,B = N,F = Y	50/69 52/64
	5	McDonald (2005), RCT	A = U,B = N,F = N	10/41 23/54
Usual care (1) vs. Education + Low/free equipment + Home safety inspection (4)	6	Kendrick (1999), Non-RCT	A = U,B = Y,F = N	214.26/323.61^a^ 223.15/323.61^a^
Usual care (1) vs. Education + Low/free equipment + Fitting (5)	7	Watson (2005), RCT	A = U,B = N,F = Y	328/718 408/742
Usual care (1) vs. Education + Low/free equipment + Fitting + Home safety inspection (6)	8	Phelan (2010), RCT	B = N,F = N,C = Y	78/147 131/146
Education (2) vs. Education + Low/free equipment (3)	9	Posner (2004), RCT	A = U,B = Y,F = Y	25/47 28/49
Education (2) vs. Education + Low/free equipment + Fitting (5)	10	Sznajder (2003), RCT	A = N,B = N,F = Y	45/50 44/47
Education + low/free equipment (3) vs. Education + low/free equipment + Home safety inspection (4)	11	Gielen (2002), RCT	A = Y,B = N,F = Y	12.85/47.44^a^ 10.87/47.44^a^
Education + Low/free equipment + Home safety inspection (4) vs. Education + Home safety inspection (7)	12	King (2001), RCT	A = Y,B = Y,F = N	158/482 166/469

Last column includes the number of households that possessed stair gates out of the total number of households

*Abbreviations*:

1.*A* Adequate allocation concealment, *B* Blinded outcome assessment, *C* The prevalence of confounders does not differ by more than 10% between treatment arms, *CBA* Controlled before-and-after study, *F* At least 80% participants of followed up in each arm, *NMA* Network meta-analysis, *RCT* Randomised clinical trial, *U* Unclear, *Y* Yes, *N* No, *NR* Not reported/not relevant

2.^a^Figures adjusted for the effect of clustering using ICC and method reported in Hubbard et al. 2014 [9]

The interventions compared across these studies in the NMAs were:

1. Usual care (UC)
2. Education (E)
3. Education + Free/low cost equipment (E + FE)
4. Education + Free/low cost equipment + Fitting (E + FE + F)
5. Education + Free/low cost equipment + Home safety inspection (E + FE + HSI)
6. Education + Free/low cost equipment + Fitting + Home safety inspection (E + FE + F + HSI)
7. Free/low cost equipment (FE only) (Poison prevention) or Education + Home Safety Inspection (E + HSI) (Falls prevention)

**Fig. 1:**
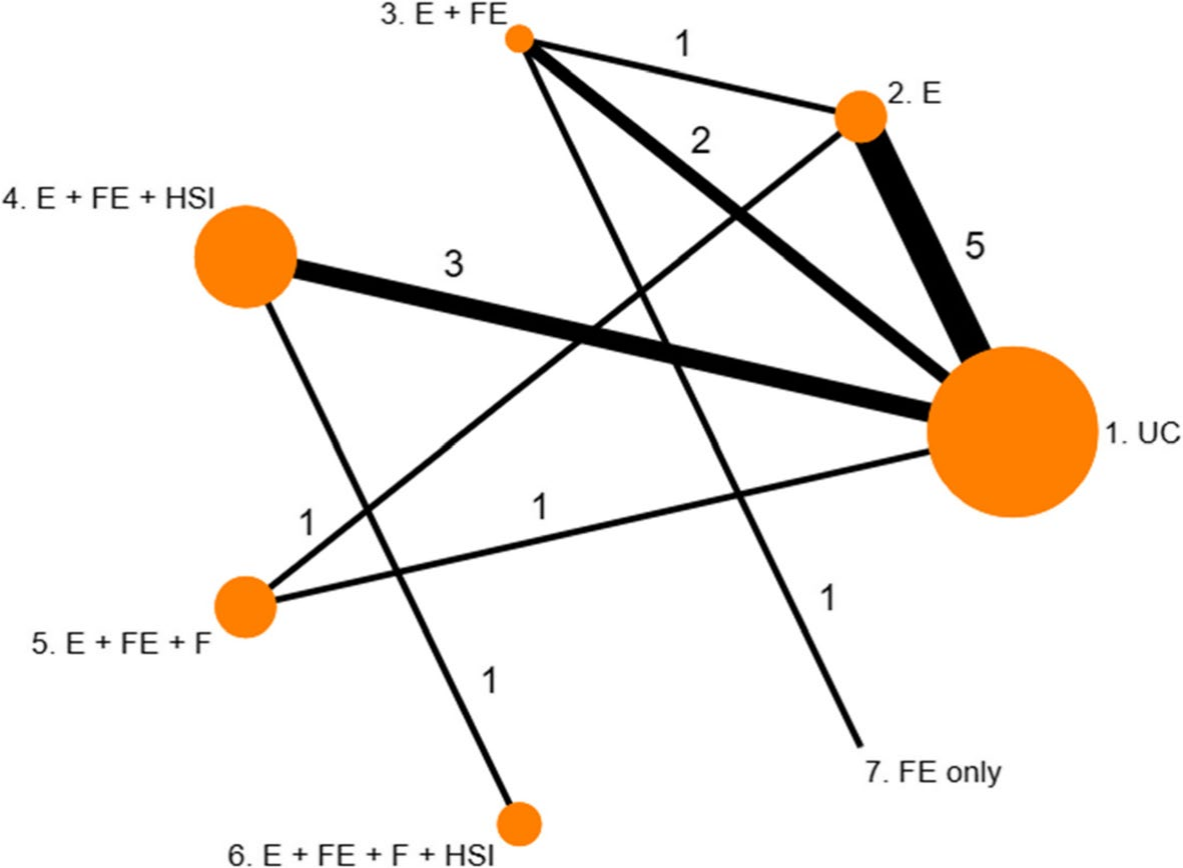
Network of interventions to prevent poisonings in the home of children aged 0–5

**Fig. 2:**
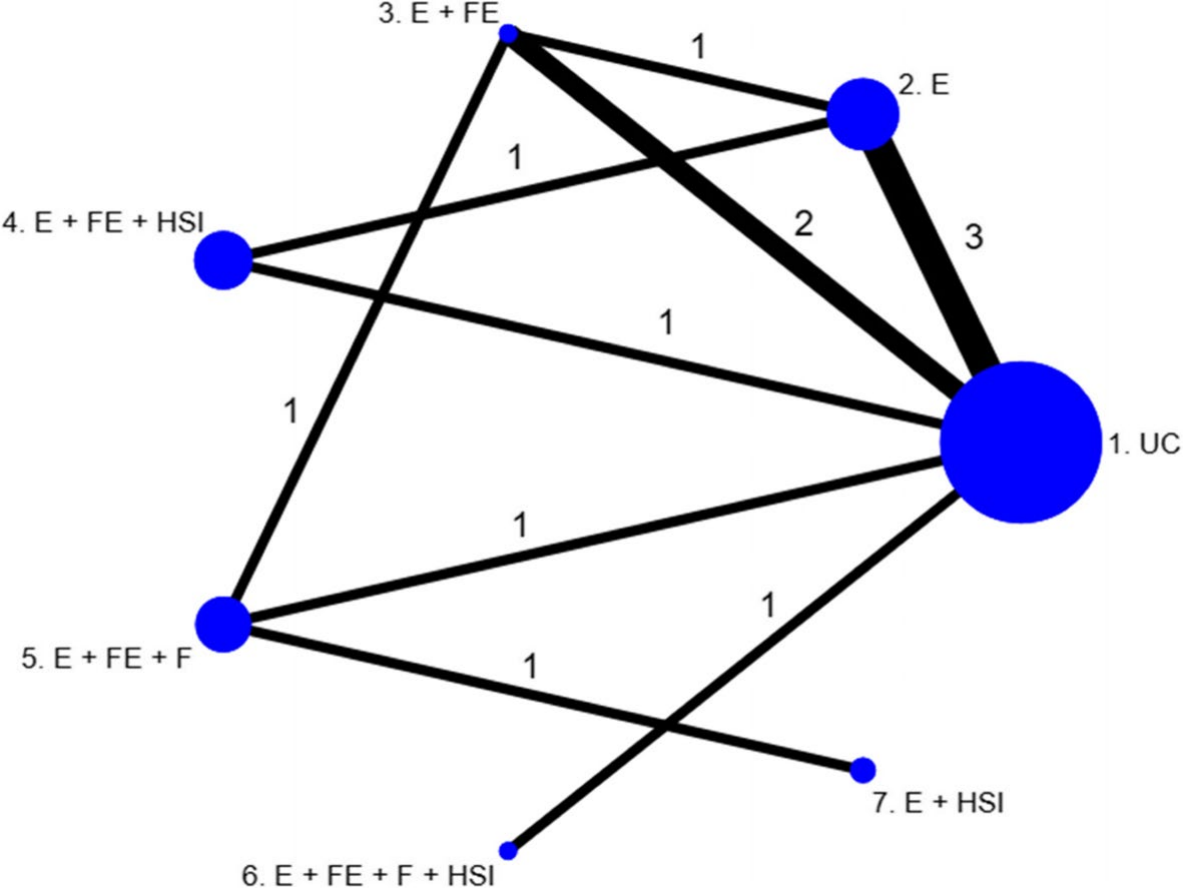
Network of interventions to prevent falls in the home of children aged 0–5

The network plots showing the comparisons between interventions for each outcome can be seen in Fig. 1 and Fig. 2.

## Results

### Safe storage of other household products

#### Network meta-analysis (NMA)

The results from the replicated published NMA can be seen in Table 3, listing the relative effects of all interventions present in the network. The results were consistent with those from the published NMA by Achana et al. [8]. Similar to Achana et al. [8], there were no issues with model fit and the between-study heterogeneity highlighted high-levels of heterogeneity. However, this was anticipated due to the low number of studies contributing direct evidence to some pairwise comparisons. Node-splitting was used to check consistency in closed loops of evidence where there was direct and indirect evidence such that there was no signs of inconsistency in the network. The relative effectiveness of the interventions are presented as odds ratios (ORs) with 95% credible intervals. From Table 3, we can see that most interventions are more effective at increasing the uptake of the poison prevention behaviours for the safe storage of other household items than *usual care*, apart from the *free/low-cost equipment* intervention. Using the results of the NMA, we ranked the interventions according to which was the most effective at increasing the uptake of the poison prevention measures in the home. The results from the rankings can be seen in Table 4**.**

**Table 3:** Results of NMA random effects model for the safe storage of household products outcome

Odds Ratio (95% Credible Interval)	Usual Care (UC)	Education (E)	Education + Free/low cost equipment (E + FE)	Education + Free/low cost equipment + Fitting (E + FE + F)	Education + Free/low cost equipment + Home safety inspection (E + FE + HSI)	Education + Free/low cost equipment + Fitting + Home safety inspection (E + FE + F + HSI)	Free/low-cost equipment (FE only)
Usual Care (UC)		1.26 (0.67, 2.46)	2.24 (0.97, 5.62)	2.514 (0.46, 4.31)	1.33 (0.46, 4.31)	2.56 (0.57, 15.15)	0.37 (0.00, 19.63)
Education (E)	1.40 (0.81, 2.60)		1.78 (0.67, 4.75)	2.00 (0.72, 6.69)	1.06 (0.35, 3.45)	2.04 (0.40, 13.37)	0.29 (0.00, 15.25)
Education + Free/low cost equipment (E + FE)	1.99 (0.45, 8.04)	2.58 (1.12, 5.94)		1.122 (0.3438, 4.325)	0.59 (0.15, 2.41)	1.15 (0.20, 8.29)	0.17 (0.00, 7.36)
Education + Free/low cost equipment + Fitting (E + FE + F)	1.18 (0.96, 1.46)	1.41 (0.49, 4.05)	NA		0.53 (0.12, 2.07)	1.03 (0.28, 4.21)	0.14 (0.00, 7.94)
Education + Free/low cost equipment + Home safety inspection (E + FE + HSI)	2.98 (0.59, 16.94)	NA	NA	NA		1.93 (0.28, 15.18)	0.27 (0.00, 16.05)
Education + Free/low cost equipment + Fitting + Home safety inspection (E + FE + F + HSI)	NA	NA	NA	NA	1.04 (0.81, 1.34)		0.14 (0.00, 9.78)
Free/low-cost equipment (FE only)	NA	NA	0.32 (0.01, 7.96)	NA	NA	NA	

The upper triangle contains the results from the NMA, and the lower triangle contains the results from a random effects pairwise meta-analysis

**Table 4:** Table of the ranking of interventions for the safe storage of other household products outcome

Intervention		Ranking (95% Credible Interval)	Probability intervention is the best
1	**Usual care (UC)**	6 (4, 7)	0.00
2	**Education (E)**	5 (2, 7)	0.01
3	**Education + Free/low cost Equipment** **(E + FE)**	3 (1, 6)	0.22
4	**Education + Free/low cost Equipment + Fitting** **(E + FE + F)**	2 (1, 5)	0.22
5	**Education + Free/low cost Equipment + Home safety inspection** **(E + FE + HSI)**	4 (1, 7)	0.05
6	**Education + Free/low cost Equipment + Fitting + Home safety inspection** **(E + FE + F + HSI)**	2 (1, 7)	0.37
7	**Free/low-cost equipment only** **(FE only)**	7 (1, 7)	0.13

From Table 4, we can see that the intervention with the highest probability of being the most effective is *education* + *free/low-cost equipment* + *fitting* + *home safety inspection (E* + *FE* + *F* + *HSI)*, which is the most intensive intervention. This intervention was also ranked highest along with *education* + *free/low-cost equipment* + *fitting (E* + *FE* + *F)*. The least effective interventions were *usual care* and *free/low-cost equipment* only. There was overlap between the 95% credible intervals for the rankings for all the interventions, indicating that no distinct intervention is optimal or worst.

#### Study level threshold analysis

**Fig. 3:**
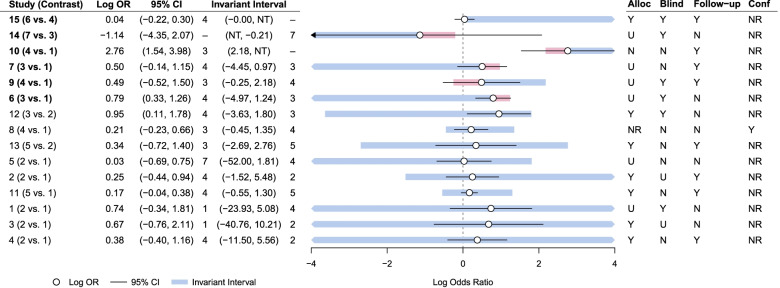
Study level forest plot for the safe storage of other household products outcome

Figure 3 presents the results of the study level threshold analysis. We can see that of the 15 studies included in the network meta-analysis, 7 studies had 95% confidence intervals extending beyond the invariant interval (indicated in bold). This demonstrates that the intervention recommendations are sensitive to the amount of imprecision in the study estimates in studies: 6, 7, 9, 10, 12, 14, and 15. For example, for study 15, which compared interventions 4 and 6, the estimated log OR of 0.04 had an invariant interval of (0.00, NT). This indicates that a change of -0.04 in the log OR would change the optimal intervention recommendation from intervention 6 to intervention 4. The NT in the upper invariant interval represents "No threshold", which illustrates that no amount of change in this direction would change the optimal intervention recommendation. For study 10, which compared interventions 1 and 4, the estimated log OR of 2.76 has an invariant interval of (2.19, 50.88). This illustrates that a change in the log OR of -0.57 is substantial enough to change the intervention recommendation from intervention 7 to intervention 3. Therefore, a change in the log odds ratio of 0.82 would change the intervention recommendation to intervention 3 being the most optimal rather than intervention 6. However, for studies 6 and 12, the upper limits of the invariant intervals lie very close to the upper limits of the 95% confidence intervals. For the remaining 8 studies, their relative 95% confidence intervals fall within the invariant intervals, which indicates that the magnitude of change required to alter the recommendation would need to be unrealistically large and, therefore, the decision is robust to plausible changes to the effect estimates for these studies.

#### Contrast level threshold analysis

**Fig. 4:**
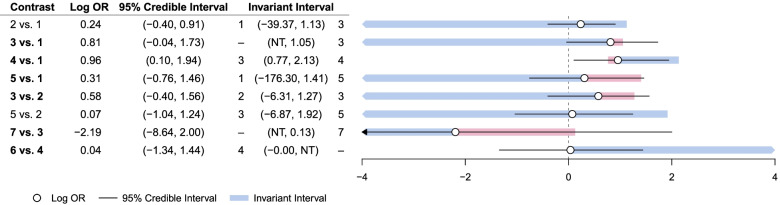
Contrast level threshold analysis for safe storage of other household products outcome

Figure 4 shows the results from the contrast level threshold analysis. Five of the intervention contrasts in the network have either upper or lower portions of their respective invariant intervals outside of the 95% credible intervals, indicating that the decision for these contrasts are sensitive to the level of imprecision in these estimates. For the other two contrasts in the network (2 vs 1, 5 vs 2), the invariant intervals are wide and contain the 95% credible interval for each estimate. This indicates that the average effectiveness estimates for these comparisons are robust to any changes in the evidence. The results from Fig. 4 are consistent with those depicted in the study level threshold analysis (Fig. 3).

It is important to note that when only one study observes a particular contrast in the network, the results of the threshold analyses at study level and contrast level must be consistent. From Fig. 1, there are two two-arm studies in the network, which are single studies for comparisons 7 vs 3 and 6 vs 4. From Fig. 4, we can see that the thresholds for the contrast 6 vs 4 are identical to those corresponding to study 15 in the study level analysis (as seen in Fig. 3), as expected. However, we can see that the 95% credible interval for the effect estimate is wider in the contrast level analysis than the 95% confidence interval in the study level analysis. This is due to the combined NMA result being less precise than the study estimate due to the large level of heterogeneity in the NMA. However, for the 7 vs 3 contrast, both the effect estimates and thresholds are different at the study level and the contrast level. Despite the quantitative differences between the study level and the contrast level analyses for this comparison, the results for this particular contrast/study are consistent qualitatively. There is a lot of uncertainty around the effect estimate for this contrast/study, and the upper threshold (in favour of intervention 7) lies well within the confidence interval at study level and credible interval at contrast level.

### Possession of a fitted stair gate outcome

#### Network meta-analysis

The results from the replicated published NMA by Hubbard et al. [9] can be seen in Table 5. The results obtained from the replicated NMA were consistent with those from the original NMA [9]. Similarly to the other NMA by Achana et al. [8], model fit and inconsistency in the network were assessed and no issues were identified. From Table 5, we can see that all interventions were effective at increasing the possession of a fitted stair gate compared to *usual care*. Using the results from the NMA, we then ranked the interventions according to which is most effective. The intervention rankings can be seen in Table 6.

**Table 5:** Results of NMA random effects model for the possession of a fitted stair gate outcome

Odds Ratio (95% Credible Interval)	Usual Care (UC)	Education (E)	Education + Free/low cost equipment (E + FE)	Education + Free/low cost equipment + Home safety inspection (E + FE + F)	Education + Free/low cost equipment + Fitting (E + FE + HSI)	Education + Free/low cost equipment + Fitting + Home safety inspection (E + FE + F + HSI)	Education + Home safety inspection (E + HSI)
Usual Care (UC)		1.45 (0.91, 2.55)	1.64 (0.91, 3.05)	1.29 (0.70, 2.87)	1.53 (0.84, 3.50)	7.85 (2.97, 21.04)	1.44 (0.54, 4.80)
Education (E)	1.53 (0.72, 4.00)		1.14 (0.56, 2.16)	0.90 (0.40, 2.10)	1.07 (0.49, 2.44)	5.45 (1.71, 15.73)	1.01 (0.32, 3.33)
Education + Free/low cost equipment (E + FE)	1.98 (0.71, 5.32)	1.17 (0.09, 5.94)		0.79 (0.38, 1.81)	0.94 (0.41, 2.54)	4.82 (1.50, 14.93)	0.89 (0.31, 2.93)
Education + Free/low cost equipment + Home safety inspection (E + FE + HSI)	1.15 (0.39, 4.31)	NA	0.78 (0.05, 11.4)		1.19 (0.45, 3.25)	6.05 (1.69, 18.76)	1.13 (0.48, 2.58)
Education + Free/low cost equipment + Fitting (E + FE + F)	1.44 (0.55, 4.44)	1.71 (0.09, 34.05)	NA	NA		5.10 (1.39, 15.55)	0.94 (0.26, 3.44)
Education + Free/low cost equipment + Fitting + Home safety inspection (E + FE + F + HSI)	7.90 (2.01, 31.4)	NA	NA	NA	NA		0.18 (0.05, 0.86)
Education + Home safety inspection (E + HSI)	NA	NA	NA	NA	1.12 (0.08, 13.66)	NA	

The upper triangle contains the results from the NMA, and the lower triangle contains the results from a random effects pairwise meta-analysis

From Table 6, we can see that the most effective intervention at increasing the possession of a fitted stair gate was *education* + *free/low cost equipment* + *fitting* + *home safety inspection*, as this intervention was ranked highest. The least effective intervention was identified as *usual care* as this intervention ranked last and had the lowest probability of being the optimal intervention. As the 95% credible intervals for all of the other interventions overlap, we cannot be certain as to where the other interventions rank according to their relative effectiveness.

**Table 6:** Table of the ranking of interventions for the possession of a fitted stair gate outcome

Intervention		Ranking (95% Credible Interval)	Probability intervention is the best
1	**Usual care (UC)**	7 (4, 7)	0.000
2	**Education (E)**	4 (2, 7)	0.001
3	**Education + Free/low cost Equipment** **(E + FE)**	3 (2, 6)	0.004
4	**Education + Free/low cost Equipment + Fitting** **(E + FE + F)**	4 (2, 7)	0.008
5	**Education + Free/low cost Equipment + Home safety inspection** **(E + FE + HSI)**	5 (2, 7)	0.002
6	**Education + Free/low cost Equipment + Fitting + Home safety inspection** **(E + FE + F + HSI)**	1 (1, 2)	0.969
7	**Education + Home safety inspection (E + HSI)**	4 (2, 7)	0.015

#### Study level threshold analysis

**Fig. 5:**
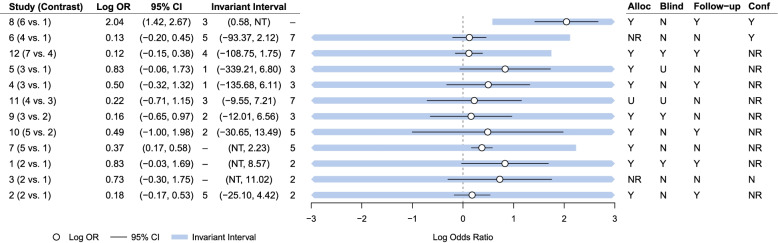
Study level forest plot for the possession of a fitted stair gate outcome

From Fig. 5, we can see that none of the invariant intervals for any of the study level effect estimates are red, which indicate that all of the 95% confidence intervals for the effect estimates lie well within the invariant intervals. This indicates that no amount of feasible change in the effect estimates would result in an alternative intervention being identified as optimal. Therefore, this highlights that the intervention recommendation from this NMA is robust to any possible changes in the evidence that could be due to any potential bias.

#### Contrast level threshold analysis

**Fig. 6:**
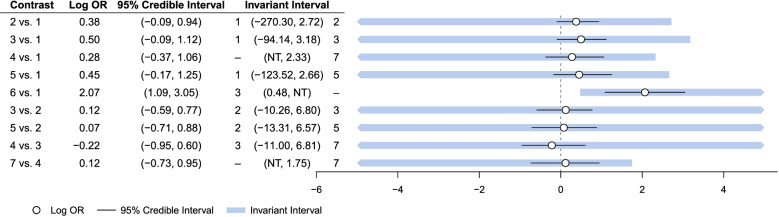
Contrast level threshold analysis for possession of a fitted stair gate outcome

As we can see in Fig. 6, all of the 95% credible intervals for the average effect estimates from each of the intervention contrasts present in the network are contained within their respective invariant intervals. Therefore, we can say that the intervention recommendation from the network is robust.

## Discussion

In both network meta-analyses, the most intensive intervention was identified as the most effective. The *usual* care intervention was identified as the least effective intervention in both of the networks, along with *free/low-cost equipment* interventions which was also identified as being least effective in the poison prevention network. For the poison prevention network, the results indicate that no distinct intervention could be recommended as the most optimal intervention, this is also illustrated in the study and contrast level threshold analysis. In the NMA, this is inferred by the credible intervals for the effect estimates and the overlapping intervention rankings. In the threshold analysis, this was reflected in the small thresholds identified in the analyses, which meant that a small change in the evidence would result in an alternative intervention being most effective. Furthermore, the intervention recommendation from the poison prevention NMA was not robust, as the effect estimate was sensitive to the level of imprecision in the evidence and potential bias. On the contrary, for the falls prevention network, there was a distinct intervention that could be recommended from the NMA, and the threshold analysis identified this recommendation as robust.

As recommended by Phillippo et al. 2018 [4], any studies with reasonably small thresholds need to be assessed for risk of bias by using the tools discussed previously. From the threshold analysis applied to the poison prevention network, there were 3 studies with thresholds less than 0.5; these were studies 6, 7 and 15. By referring to the study quality assessment in Table 2, these studies did not appear to be particularly at risk of bias and did not have any major issues with their quality.

A limitation of this work is that the published NMA examples only had a small number of studies contributing to each of the networks. There was little evidence for many of the intervention contrasts. As well as this, there was no distinct or clear intervention recommendation from the poison prevention NMA as all effect estimates contained 1, and the rankings overlapped. However, this example still illustrates the use of NMAs and threshold analysis in the context of public health and highlights that any recommendations from this example are not robust.

Threshold analysis allows researchers to identify and quantify the robustness of intervention recommendations from NMAs to any potential bias in the evidence. The use of this method provides researchers and policy makers with the confidence that their results from NMAs are robust to changes in the evidence that might be due to potential risk of bias or imprecision. It is important to note that threshold analysis does not investigate the presence or absence of any particular bias and does not make any assumptions on the type and source of the bias. Threshold analysis is more concerned with the implications, if there is any bias present, that such bias would have on the intervention recommendations and resulting decisions [4, 12].

There are several other tools available to assessing the quality of network meta-analyses and their results. The Grading of Recommendations Assessment, Development and Evaluation (GRADE), also formerly known as GRADE NMA, has been developed to assess the quality of evidence contributing to the intervention contrasts for every pair of interventions. The quality of evidence for each contrast in the network is rated as high, moderate, low, or very low across five areas: inconsistency, study limitations, indirectness, imprecision, publication bias. However, as networks become larger, loops of evidence become more complex leading to GRADE NMA becoming insufficient [6].

Another example of a tool to assess the quality of NMAs, is the recently developed CINeMA (Confidence in Network Meta-analysis) which is accompanied by user-friendly software. CINeMA, unlike GRADE NMA, can be used for any type of network [13]. Both GRADE NMA and CINeMA consider the plausibility of assumptions but do not give numerical indication of the certainty of recommendations from NMAs, which could be more useful for decision-makers and guideline developers [6]. However, we are not stating that one method here is better than the other, each method/tool has its own advantages and disadvantages and have different aims, which should be considered at the users own discretion.

Threshold analysis could be extended to incorporate GRADE judgements in the analyses, as seen in the paper by Holper 2019 [14]. The use of GRADE judgements alongside threshold analysis offers a qualitative judgement as well as quantitative. Threshold analysis could also be incorporated into a cost-effectiveness analysis to consider the robustness of decisions on the cost-effectiveness of interventions.

A further application of threshold analysis could be to components network meta-analysis. Component network meta-analysis expands on the NMA framework and splits the interventions into components to consider which combination of components is most effective. The interventions in the NMA assessed in this example consist of several components, for example, education, fitting, and home safety inspection, so it could be more appropriate to explore which combinations of these components, not just the ones observed, are most effective. As well as this, in recent literature, threshold analysis has been applied to continuous and binary outcomes. These methods could be extended to look at other possible outcomes.

There still should be some careful consideration when applying complex evidence synthesis methods to highly heterogeneous data, as threshold analysis is not a way to fix the issues that arise. The primary consideration with heterogeneity is that we should account for it appropriately rather than avoid complex analyses due to the arising issues. Heterogeneity is inevitable, especially in public health intervention appraisals. The use of advanced methods for evidence synthesis, including the appropriate account of the heterogeneity, can lead to more detailed and robust conclusions, which will improve research and aid the decision-making process [3].

## Conclusion

Applying threshold analysis to two NMAs of public health interventions, we have highlighted the use of threshold analysis to identify when an intervention recommendation is robust to any possible changes in the evidence due to potential bias, and when the recommendation is not robust. We have illustrated that threshold analysis gives an insight into the effects of possible changes in the evidence on the resulting intervention decisions from NMAs. The application of threshold analysis should ease any hesitancy to use complex evidence synthesis methods, such as NMA, in public health intervention appraisals. The increase in the use of such methods in public health intervention appraisals can improve the standard of the evaluation of interventions and, consequently, the decision-making process, with benefit to policy-makers and the public.

## Data Availability

The dataset supporting the conclusions of this article is included within the article.

## Abbreviations

NICE: National institute for health and care excellence
NMA: Network meta-analysis
RCT: Randomised controlled trial
NRCT: Non-randomised controlled trial
GRADE: Grading of Recommendations Assessment, Development and Evaluation
UC: Usual care
E: Education
FE: Free/low cost equipment
F: Fitting
HSI: Home safety inspection
OR: Odds ratio
CrI: Credible interval
CI: Confidence interval
